# Application of a reverse stethoscope to overcome communication barriers: A case report of an elderly patient with illiteracy and profound hearing loss

**DOI:** 10.1097/MD.0000000000045546

**Published:** 2025-10-24

**Authors:** Qin Zhang, Hongqun Zhang, Xiaojuan Han, Zhuoyin Wang, Qi Chen

**Affiliations:** a Department of Blood Purification Center, Ningbo Beilun Third People’s Hospital, Ningbo, Zhejiang, China; b Department of Nursing Department, Ningbo Beilun Third People’s Hospital, Ningbo, Zhejiang, China; c Department of ICU, Ningbo Beilun Third People’s Hospital, Ningbo, Zhejiang, China; d Department of Surgery, Ningbo Beilun Third People’s Hospital, Ningbo, Zhejiang, China; e Department of Surgery, Ningbo Mingzhou Hospital, Ningbo, Zhejiang, China.

**Keywords:** hearing loss, reverse stethoscope technique, stethoscope

## Abstract

**Rationale:**

As a common clinical diagnostic tool, stethoscopes have the ability to amplify sound, isolate ambient noise, and locate the source of sound. However, we reversed the stethoscope innovatively. This changed its standard use, delivering sound directly to the patient’s ear. It successfully overcame communication barriers between clinicians and patients who have both illiteracy and severe hearing loss (HL).

**Patient concerns:**

This paper reports a case of a 74-year-old elderly male patient with illiteracy combined with very severe HL requiring a hearing decibel of 82 dB, living alone, who visited our hemodialysis unit because of end-stage renal failure requiring hemodialysis treatment.

**Diagnoses:**

Uremia, Chronic kidney disease stage 5, Hearing loss.

**Interventions:**

Reverse stethoscope technique using a stethoscope between doctor and patient.

**Outcomes:**

Communication barriers were addressed and the physician successfully took a medical history.

**Lessons:**

For patients with illiteracy combined with very severe HL, we can interrogate them with a stethoscope reverse auscultation technique.

## 1. Introduction

Effective communication between healthcare providers and patients is the cornerstone of clinical diagnosis and treatment. However, this process is often particularly challenging for patients with hearing and language impairments. According to the World Health Organization report Deafness and HL, over 25% of people aged ≥60 have disabling hearing loss (HL). The prevalence of disabling HL rises exponentially with age. HL not only has a physiological impact, but it also significantly affects patients” psychological well-being, social interactions, and overall quality of life. Impairments lead to communication difficulties, with elderly individuals struggling to follow conversations – especially in noisy environments – which in turn hinders effective communication with family, friends, and healthcare providers.^[1–4]^

The consequences of HL extend beyond mere communication barriers. Reduced auditory stimulation can contribute to a decline in cognitive function, thereby accelerating the deterioration of mental abilities.^[5–9]^ Moreover, the psychological impact of HL can be profound, often exacerbating feelings of isolation and depression among the elderly.^[10–13]^ Although various rehabilitation strategies – such as auditory training, the use of hearing aids (HA), and cochlear implants – can markedly improve outcomes, their global uptake remains relatively low.^[14–19]^

China, one of the fastest aging countries in the world, faces additional challenges as the elderly population often contends with multiple communication barriers, such as illiteracy and the inability to use sign language. Traditional methods (e.g., body language, written notes, or basic voice amplification) often prove inadequate. This increases misdiagnosis risks and jeopardizes patient safety.

In this context, we present the first case report introducing the “reverse stethoscope” technique. This innovative stethoscope modification proved vital in emergency settings. It overcame communication barriers for a hemodialysis patient with profound HL and illiteracy. This case provides a potentially replicable solution for enhancing clinical communication in resource-limited environments.

## 2. Case presentation

We report a case of a 74-year-old male patient who lived alone and presented for regular hemodialysis due to end-stage renal disease. The patient suffered from profound HL – requiring a sound level of 82 dB for auditory perception – and was illiterate, recognizing only simple graphical symbols. In addition, his prolonged solitary living prevented him from acquiring basic sign language skills. He had no history of using a hearing aid or cochlear implant and, due to limited financial resources and a lack of family support, was unable to afford any assistive hearing devices.

At his initial presentation, the medical team encountered multiple challenges. First, the patient could not provide a conventional medical history; his only complaint was an ambiguous gesturing toward the lumbar region (later confirmed as lumbar pain secondary to uremic peripheral neuropathy). Second, his severe hearing impairment blocked understanding of hemodialysis procedures. This caused significant anxiety – evidenced by a heart rate of 110 bpm and respiratory rate of 24 breaths/minute. In response, the team sequentially attempted 3 traditional communication strategies: Increased Volume: The physician spoke at a distance of 30 cm using a volume exceeding 90 dB (comparable to the noise of a lawnmower). However, sound distortion rendered the message unintelligible, and communication completely failed.

Gestural Communication: The team used body language – mimicking kidney shapes and puncture procedures – but the patient failed to understand these gestures and subsequently refused further evaluation and treatment. Text/Image Assistance: A simplified pictorial and textual manual (including schematic diagrams of the dialysis machine and cartoon depictions of the treatment process) was used; however, due to the patient’s illiteracy and the abstract nature of the images (for example, using red arrows to indicate “risk”), the intended messages were not effectively conveyed. In the baseline evaluation, physicians employed 10 standardized questions (e.g., “Do you have chest pain?” and “Has your urine output decreased?”) to assess communication efficiency. Initially, the patient answered only 1 question correctly (10% accuracy). Average response time reached 4.5 minutes per question. The total interview lasted 45 minutes. Frustrated by these repeated communication failures, the patient made several attempts to leave the facility. Due to the lack of hearing assistive equipment in our outpatient setting – a scenario common in Chinese secondary hospitals – the team decided to employ a reverse stethoscope technique.

Reverse Stethoscope Technique A standard dual-ear stethoscope was prepared, ensuring that both the earpieces and the diaphragm surface were clean and in good working order. The earpieces were gently inserted into the patient’s external auditory canals at a 30-degree angle to avoid causing discomfort from excessive pressure. Next, the physician held the chest piece approximately 2 cm in front of his mouth and spoke at a normal volume (60–70 dB, which is typical for indoor conversation). Emphasis was placed on key words such as “dialysis,” “pain,” and “medication” by prolonging the syllables (approximately 0.5 seconds each). This method harnessed the resonant properties of the stethoscope’s tubing to enhance the transmission of low-frequency sound waves (125–500 Hz). Sound waves traveled directly through the rubber tubing into the patient’s ear. Bypassing the diseased outer and middle ear structures (which may include issues such as tympanic membrane sclerosis or ossicular fixation) and stimulating the cochlear hair cells via bone conduction.

To verify the effectiveness of this innovative approach, the same set of 10 standardized questions was administered again. After intervention, accuracy reached 80% – 8 of 10 questions answered correctly. The remaining errors primarily resulted from dialect-Mandarin miscommunication in southern regions. Time Efficiency: The total interview duration was reduced to 12 minutes, averaging 1.2 minutes per question. Physiological Improvement: Anxiety-related vital signs normalized, with the patient’s heart rate decreasing to 78 beats per minute and respiratory rate to 16 breaths per minute. Subjective Feedback: Assessment using a visual analog scale revealed an increase in patient satisfaction from 3/10 to 8/10. The patient even expressed his gratitude by shaking the physician’s hand.

**Figure 1.**
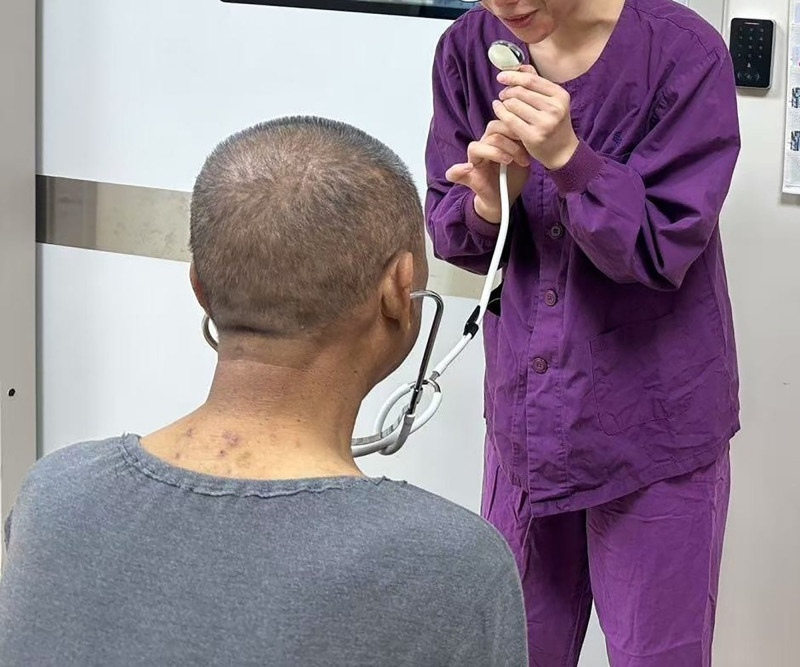
Application of the reverse stethoscope technique for communication with a hearing-impaired and illiterate patient. Note: The physician is shown speaking into the diaphragm of the stethoscope while the earpieces are placed in the patient’s ears. This method allows direct transmission of speech sounds through the stethoscope tubing, bypassing the patient’ impaired outer and middle ear structures and facilitating effective communication despite profound hearing loss and illiteracy.

During the treatment process, an “enhanced informed consent” protocol was also implemented. A nurse demonstrated the hemodialysis puncture procedure while the physician – using the reverse stethoscope technique – explained the associated risks and benefits step by step. The patient subsequently signed the informed consent form. This case report was approved by our hospital’s ethics committee, and all patient-identifying information has been anonymized (Fig. 1).

## 3. Discussion

HL is a pervasive yet often hidden phenomenon among the elderly, particularly in developing countries and regions. According to the World Health Organization report Ageing and Health in China, the population aged 60 and above in China reached 254 million by the end of 2019, and is projected to increase to 402 million by 2040. The rapid expansion of the elderly demographic accentuates age-related health issues – such as HL, cognitive decline, and high illiteracy rates. Indeed, most Chinese individuals aged 60 and above exhibit some degree of hearing impairment, with the incidence of profound HL rising annually.

The most common interventions for HL involve the use of HA or cochlear implants; however, their adoption rates remain relatively low on a global scale.^[18,19]^ For example, in the United States, it is estimated that only 14.2% of adults aged 50 and older with a HL of 25 dB HL or greater use HA.^[20]^ In Australia, an earlier study by Chia et al^[21]^ reported that only 25.5% of hearing-impaired individuals regularly use HA. In China, data from the 2021 Report on Hearing Health in China indicate that only 6.5% of the elderly population utilizes HA. Several factors may contribute to this low usage rate. First, many elderly individuals regard HL as an inevitable consequence of aging and do not consider it worthy of intervention.^[15,22,23]^ Second, a lack of adequate knowledge about HA^[24]^ combined with perceptions of high cost discourages their use. Furthermore, psychological barriers exist – some elderly patients, as noted by Ruusuvuori et al,^[25]^ view wearing a hearing aid as a form of age discrimination, evoking feelings of shame. The complexities involved in tuning and maintaining HA^[26,27]^ and the high cost and postoperative demands of cochlear implants^[8,28]^ further diminish their uptake, even when some regions have begun to offer partial health insurance coverage.

The communication challenges faced by elderly patients with HL become especially pronounced in clinical settings. Traditional patient interviews rely heavily on verbal communication, which is often ineffective for these individuals. Many patients report that the noisy environment of medical facilities and the lack of adequate visual support impede successful communication – even rendering it nearly impossible.^[29]^ In many Chinese healthcare settings, the absence of dedicated communication aids (such as hearing devices) and the occasional failure of patients to bring their own further exacerbate these issues. Consequently, poor communication may lead to decreased service quality and increased economic burdens through higher medical costs and longer treatment times.^[30]^ In emergent situations, communication barriers may delay patient assessment and treatment, adversely affecting clinical outcomes. Communication barriers prevent elderly patients from understanding medical instructions. This heightens psychological distress, causing anxiety and helplessness. Some may even resist treatment. Thus, an effective communication strategy is essential for ensuring that patients understand and adhere to medical recommendations, reducing stress, and ultimately improving overall health outcomes.^[31]^

Traditionally, the stethoscope is employed as an indispensable diagnostic tool to amplify sound, isolate ambient noise, and localize physiological signals such as heart and lung sounds. Its potential as a communication device, however, remains largely unexplored. The innovative reverse application of the stethoscope represents a novel approach to address communication challenges in patients with HL. By repurposing a standard stethoscope to transmit sound directly to the patient’s ear, physicians can quickly bridge communication gaps and more effectively ascertain patient needs.^[32]^ This approach is not only simple and noninvasive but also does not require any additional equipment – making it readily available in hospital settings as a temporary communication aid. Especially for patients who are both illiterate and profoundly hearing impaired.

The innovation presented in this case extends beyond technical modifications; it also highlights the urgent need for adaptive clinical solutions in resource-limited environments. Conventionally, the stethoscope is designed as a passive device for capturing internal body sounds. Here, through a reverse-engineering approach, the stethoscope has been transformed into an active sound-emitting device. Its underlying mechanism leverages the stethoscope’s enclosed tubing system to form an acoustic resonant cavity. When the physician speaks into the device, sound waves are repeatedly reflected within the tubing. Low-frequency components – less attenuated due to longer wavelengths – accumulate and amplify output at the earpiece.

The clinical value of this technique is underscored in 3 dimensions: Immediate Accessibility: In primary care settings or resource-poor hospitals, HA are often scarce. Stethoscopes – as standard-issue equipment – can be readily repurposed into communication tools. This requires no added cost or maintenance. Cross Departmental Applicability: Emergency Department: For patients experiencing transient HL following trauma, the reverse stethoscope can convey critical instructions (e.g., “do not move your neck”) directly. Pediatrics: By incorporating playful elements with toy stethoscopes, this technique may reduce procedural anxiety in children with hearing impairments. Geriatrics: It can be used for cognitive orientation training in elderly patients with conditions such as Alzheimer disease (for example, by repeating time and place information daily). Cultural Adaptability: In regions with pronounced dialectical variations (such as Southern China), physicians could record and playback messages in the local dialect via the stethoscope. Thereby minimizing misunderstandings stemming from nonstandard Mandarin pronunciation.

Nevertheless, this technique is not without limitations. Its efficacy depends on the structural integrity of the patient’s external auditory canal; individuals with narrow ear canals or cerumen impaction may not achieve effective acoustic coupling. Additionally, high-frequency components of speech (such as consonants like/s/ and/f/ that exceed 2000 Hz) tend to be significantly attenuated in the tubing, potentially leading to misinterpretation. Prolonged use of this method might also cause irritation or secondary infections of the external auditory canal.

From a health systems perspective, this case underscores existing deficiencies in accommodating patients with sensory impairments. According to a 2022 report by the China Disabled Persons’ Federation, only 12% of county-level hospitals are equipped with basic hearing assistance devices, and healthcare staff generally lack training in adaptive communication strategies.

Future research should aim to validate the generalizability of the reverse stethoscope technique through multicenter studies with larger sample sizes. Additionally, there is a need to develop quantitative evaluation tools – such as voice spectrum analysis software – to objectively assess the improvements in acoustic transmission. Long-term studies are also necessary to monitor safety. Particularly regarding the effects of external auditory canal pressure changes on vestibular function and potential differences in acoustic performance among various stethoscope materials (e.g., rubber vs polyurethane).

## 4. Conclusion

Effective communication between clinicians and patients is essential for conveying information about clinical symptoms, diagnoses, and treatment plans, as well as for facilitating patient-centered shared decision-making.^[33]^ When managing patients with HL in the absence of any auxiliary hearing devices, the stethoscope – readily available in hospital settings as an amplification tool – can effectively overcome this challenge. This approach has been shown to dramatically shorten interview times, enhance communication between healthcare providers and patients, and improve overall patient satisfaction.^[34]^ For clinicians, the technique is straightforward to implement, offering a safe, high-quality, patient-centered means of care for individuals with hearing impairments.

This innovation transcends the conventional physical limitations of patient-clinician communication by providing a simple yet effective method for patients with profound HL and illiteracy. It offers an immediate solution for emergency consultations in resource-limited environments and holds broad clinical application prospects. In the future, dedicated stethoscope devices designed specifically for hearing-impaired patients, integrated with advanced voice amplification technology, may further enhance communication outcomes.

## Author contributions

**Conceptualization:** Qin Zhang, Hongqun Zhang, Xiaojuan Han, Zhuoyin Wang, Qi Chen.

**Investigation:** Qin Zhang, Hongqun Zhang, Xiaojuan Han.

**Methodology:** Qin Zhang, Zhuoyin Wang, Qi Chen.

**Supervision:** Qin Zhang, Zhuoyin Wang, Qi Chen.

**Validation:** Qin Zhang, Zhuoyin Wang, Qi Chen.

**Writing – original draft:** Qin Zhang, Zhuoyin Wang, Qi Chen.

**Writing – review & editing:** Qin Zhang, Zhuoyin Wang, Qi Chen.
